# Pattern recognition and spatial differentiation of built environment around urban sports facilities from perspective of facility hierarchy: a case study of Changsha city

**DOI:** 10.3389/fpubh.2026.1947943

**Published:** 2026-09-11

**Authors:** Guiming Zhang, Suyue Qiu, Guosheng Ding

**Affiliations:** 1 School of Architecture and Planning, Hunan University, Changsha, Hunan, China; 2 School of Economics and Management, Changsha University, Changsha, China

**Keywords:** built environment, facility hierarchy, pattern recognition, spatial differentiation, urban sports facilities

## Abstract

Under the context of healthy city development and the construction of 15-min life circles, the relationship between the built environment and residents' physical activity and health has attracted increasing attention. However, most existing studies adopt a residential-centered perspective, neglect the heterogeneity of service ranges across different levels of sports facilities, and insufficiently integrate objective built-environment characteristics with residents' facility-use behaviors and subjective perceptions. Taking Changsha city as a case study, this paper develops a hierarchical sports facility-based analytical framework for life circles and investigates the spatial differentiation and typological patterns of the surrounding built environment. Unlike fixed-buffer approaches, the framework employs level-specific measurement scales reflecting differences in potential facility service ranges and access modes. The study integrates multi-source geospatial data, including OpenStreetMap road networks, POI data, WorldPop population grids, and NDVI remote sensing data, to construct a multidimensional built environment indicator system covering accessibility, functional structure, facility supply, ecological quality, and population characteristics. Methods including GIS spatial analysis, one-way ANOVA, K-means clustering, and chi-square tests are employed to identify spatial patterns across different facility levels. Questionnaire results concerning activity-space choices, travel characteristics, facility use, and environmental perceptions are interpreted alongside the corresponding accessibility, functional, and ecological dimensions of the objective spatial analysis. This integration is conducted at the aggregate level and is not used to infer individual-level associations or causality. The results show that: (1) sports facilities in Changsha city exhibit a “large number of small-scale facilities and limited but spatially dominant large facilities” distribution pattern; (2) significant differences exist in the built environments surrounding different facility levels, with population density and POI functional diversity being the key distinguishing indicators; small facilities are mainly embedded in high-density residential areas, while large and extra-large facilities are more frequently located in areas with higher functional mix and higher mean NDVI values; (3) four typical built-environment patterns are identified, including high-density and service-intensive, low-intensity built-environment, high-NDVI and low-density, and functionally mixed and service-intensive types, showing a clear center-periphery gradient; (4) respondents mainly reported using parks, waterfront and mountain trails, and walking paths, and predominantly reached sports facilities through active travel within 15 min. These behavioral and perceptual results provide complementary context for the accessibility, functional, and ecological patterns identified by the spatial analysis, while concerns remain regarding pedestrian continuity and facility management.

## 1 Introduction

Against the backdrop of increasingly prominent global public health challenges and the continuous reshaping of urban spatial structures, understanding the relationship between the built environment and residents' participation in physical activity has become a key issue of shared concern in urban planning, public health, and geographic information science (1–3). A substantial body of research indicates that insufficient physical activity is closely associated with obesity, cardiovascular diseases, and various chronic non-communicable diseases (4). As an essential setting for daily activities, the urban spatial environment is closely associated with physical activity behaviors. With the ongoing acceleration of urbanization, understanding the spatial correspondence between built-environment conditions and residents' physical activity has become an important basis for healthy-city planning.

In recent years, the concept of the “15-minute life circle” has gained increasing attention in urban governance practices worldwide. This concept emphasizes residents' convenient access to daily necessities and public service facilities within a time threshold reachable by walking or cycling, thereby enhancing urban livability, equity in public service provision, and residents' health levels (5, 6). However, existing studies have certain limitations, where most analyses adopt residential locations as a single spatial starting point, failing to capture differences in service provision across public facilities of varying hierarchical levels and lacking a systematic characterization of built environment types and their spatial differentiation patterns. Moreover, applying a uniform buffer radius to facilities at different hierarchical levels implicitly assumes comparable service capacities, attraction ranges, and dominant access modes, despite their differentiated functions in actual urban settings. For instance, Pravalika et al. (7) proposed an evaluation framework for community built environments and public services based on 15-min walking accessibility, emphasizing the assessment of a community's capacity to support residents' daily activities through the configuration and accessibility of facilities surrounding residential locations. This approach provides an important reference for the spatial quantification of the 15-min life circle. However, the study primarily adopts a residence-centered perspective and does not sufficiently distinguish differences in service coverage and spatial attractiveness among facilities of different hierarchical levels. In addition, it pays limited attention to types of built environments and their spatial differentiation characteristics, and lacks an in-depth analysis of residents' exercise intentions and actual physical activity behaviors. Omwamba et al. (8) presented a last-mile accessibility assessment method within the framework of the 15-min city, incorporating a slope-sensitive network analysis model. This approach systematically examined differences in service accessibility under walking and cycling conditions in cities with complex terrain, and, through travel behavior surveys, revealed the relationships among topography, infrastructure, and mode choice. However, the study mainly focused on integrated public services and last-mile travel efficiency, still adopts a residence-based analytical perspective, does not distinguish the heterogeneity in service catchment areas and spatial attractiveness among facilities of different hierarchical levels, and pays relatively insufficient attention to the built environment characteristics and spatial differentiation of specific functional spaces such as sports facilities.

Meanwhile, numerous studies have examined the relationships between built-environment characteristics and physical activity and health outcomes from multiple perspectives, mainly focusing on road connectivity, land-use mix, population density, green space accessibility, and the provision of public service facilities. Existing research generally suggests that favorable walking environments, higher functional diversity, and sufficient provision of sports facilities are positively associated with the frequency and continuity of residents' participation in physical activity. However, there are still certain limitations in the perspectives and methods adopted in these studies. On the one hand, many studies construct uniform buffer zones or neighborhood units centered on residential locations and assume that different sports facilities have identical service capacities, thereby neglecting the significant differences in scale, function, and attraction range among facilities. Such a uniform-buffer approach may create a mismatch between the analytical scale and facility service range. A relatively large fixed buffer may extend beyond the primary catchment of small facilities, incorporating areas less relevant to their daily users and diluting the characteristics of their immediate surroundings. Conversely, a relatively small fixed buffer may omit population, functional, and transport conditions relevant to the broader catchments of large and extra-large facilities. Consequently, a uniform radius may mischaracterize the built environments surrounding facilities at different levels and bias cross-level comparisons. On the other hand, some studies focus more on objective spatial indicators and pay insufficient attention to residents' subjective perceptions and exercise intentions, as well as to how these correspond with objective built-environment conditions, making it difficult to characterize these relationships comprehensively. For instance, Zhang et al. (9) put forward an analytical framework based on survey data from residents in core cities of the Yangtze River Delta, integrating the built environment, physical activity, and subjective well-being to examine the relationships among these dimensions. The study reported that accessibility and safety were positively associated with physical activity and subjective well-being and identified a mediating role for physical activity. However, this study primarily relied on a uniform neighborhood-scale approach and subjective questionnaire data, neglecting differences among sports facilities in terms of scale, function, and service coverage. Wali et al. (10) developed a structural equation modeling framework based on the Rails and Health cohort data to systematically examine the relationships among the built environment, physical activity, and health costs. The study found that walking-, cycling-, and public transport-related physical activities were significantly associated with lower healthcare expenditures and reported an indirect association between walkability and health costs through physical activity. However, the study still has certain limitations, which relies heavily on uniform buffer-based representations of residential environments and assuming identical service capacity across different facilities, thereby neglecting differences in facility scale, function, and attraction range.

In real urban space, sports facilities exhibit clear hierarchical differentiation. Small-scale sports facilities are typically embedded within communities and mainly serve residents' daily and fragmented fitness needs (11). They have relatively limited service areas but high usage frequency. In contrast, large and extra-large sports facilities are often located at urban functional nodes or in areas with ecological resources, possessing stronger regional service capacity and spatial attractiveness (12–14). Their service catchment areas and usage patterns differ fundamentally from those of small facilities. Therefore, ignoring the hierarchical differences among sports facilities and adopting a uniform service radius or analytical scale may lead to biased interpretations of their spatial effects, thereby affecting the scientific validity and targeting of planning decisions. A uniform radius cannot adequately represent both the localized, walking-oriented catchments of small facilities and the broader, multimodal catchments of large and extra-large facilities. Therefore, the analytical scale should account for facility hierarchy rather than treating all sports facilities as having homogeneous service ranges.

Based on the above background and identified limitations, this study takes the main urban area of Changsha city as the research area and introduces a hierarchical perspective of sports facilities to construct a multi-source data-driven built environment analytical framework. Rather than applying a uniform buffer to all facilities, the framework aligns the spatial measurement scale with differences in potential service ranges and dominant access modes across facility levels, thereby reducing potential spatial-scale mismatch. By integrating geographic information system (GIS) spatial analysis and clustering methods, the study identifies the spatial differentiation characteristics and compositional patterns of the built environment surrounding sports facilities of different levels. The results show that significant differences exist in the surrounding environments of sports facilities across different levels, with population density and point of interest (POI) functional mix index serving as key distinguishing indicators. Small-scale facilities are mainly located in high-density residential communities, whereas large and extra-large facilities are more often situated in areas with higher functional diversity. In contrast, commercial density, sports facility density, and road density show relatively limited variation across facility levels, while the difference in NDVI is modest in absolute magnitude. These results indicate limited cross-level variation in some of the measured indicators rather than demonstrating balanced or equivalent spatial provision. The clustering results reveal four typical patterns, including the high-density and service-intensive type, low-intensity built-environment type, high-NDVI and low-density type, and functionally mixed and service-intensive type. Small facilities occur across a relatively broad range of built-environment contexts, while higher-level facilities are more frequently associated with functionally mixed and service-intensive or high-NDVI and low-density environments, showing an overall gradient from the urban center to the periphery. To integrate residents' facility-use experiences with the objective spatial findings, the questionnaire results are interpreted through corresponding built-environment dimensions. Travel modes, travel times, and walking perceptions are examined alongside accessibility characteristics; perceptions of facility functions and surrounding services are considered in relation to facility provision and POI functional mix index; and the use of waterfront, mountain, and walking trails is discussed together with NDVI and the the high-NDVI and low-density pattern. This thematic and aggregate-level interpretation places the objectively measured built-environment findings alongside residents' reported physical activity behaviors and subjective perceptions. Overall, this study characterizes the spatial differentiation between sports facility hierarchy and the surrounding built environment and provides an integrated spatial, behavioral, and perceptual perspective for optimizing the allocation of urban sports facilities.

## 2 Study area

**Figure 1:**
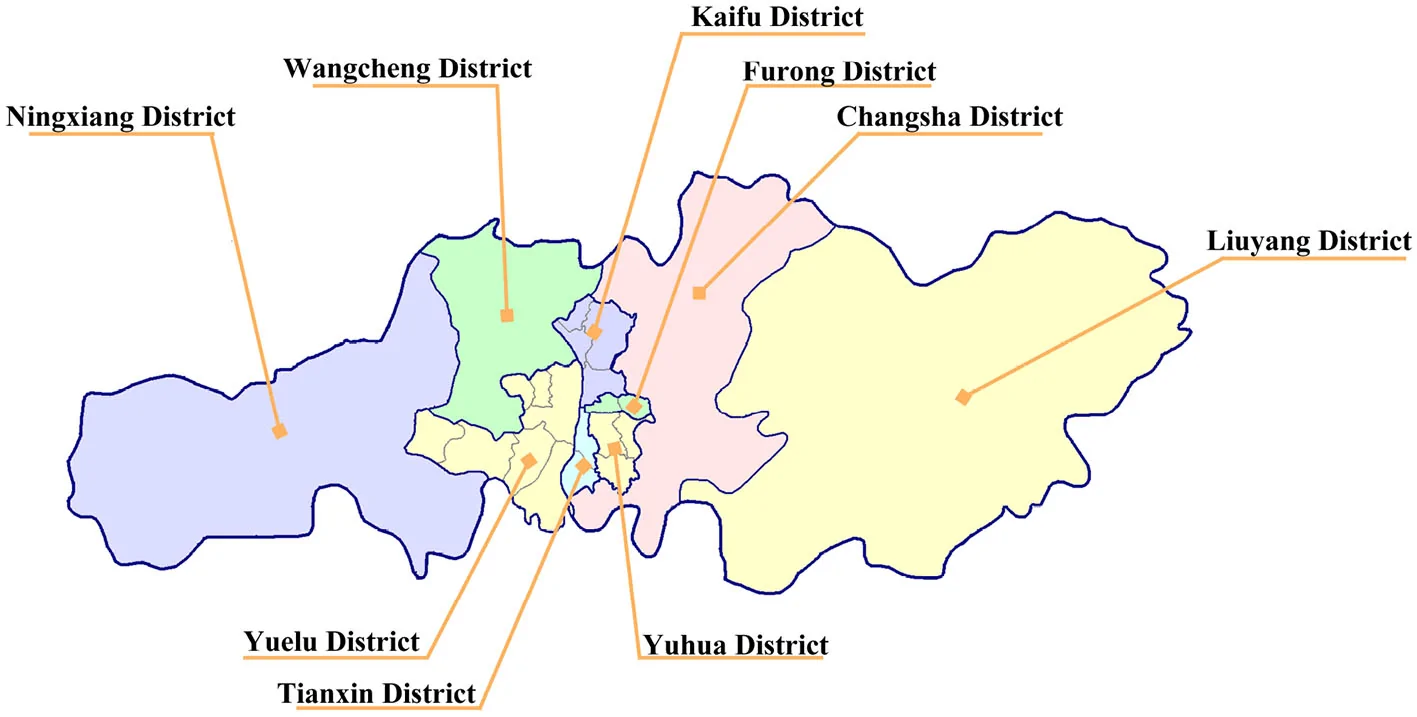
Spatial distribution of the nine districts in Changsha city.

This work selects Changsha city as the research area. As a rapidly urbanizing provincial capital in central China, Changsha city has undergone substantial population growth and spatial restructuring in recent decades, resulting in pronounced heterogeneity in its built environment. The city exhibits clear variations in population density, land-use mix, transportation networks, and ecological conditions, which provide a representative context for examining spatial differences in sports facilities and their surrounding built environments (15, 16). In addition, Changsha city has actively promoted public fitness and the development of urban open spaces, leading to a diversified system of sports facilities across different scales and service levels. These characteristics make Changsha city a suitable case for jointly characterizing sports facility hierarchy and built-environment patterns and descriptively examining residents' reported physical activity behaviors in a contemporary Chinese urban context. Currently, the Changsha city consists of nine districts: Ningxiang district, Wangcheng district, Yuelu district, Tianxin district, Furong district, Yuhua district, Kaifu district, Changsha district, and Liuyang district, as shown in Figure 1.

**Figure 2:**
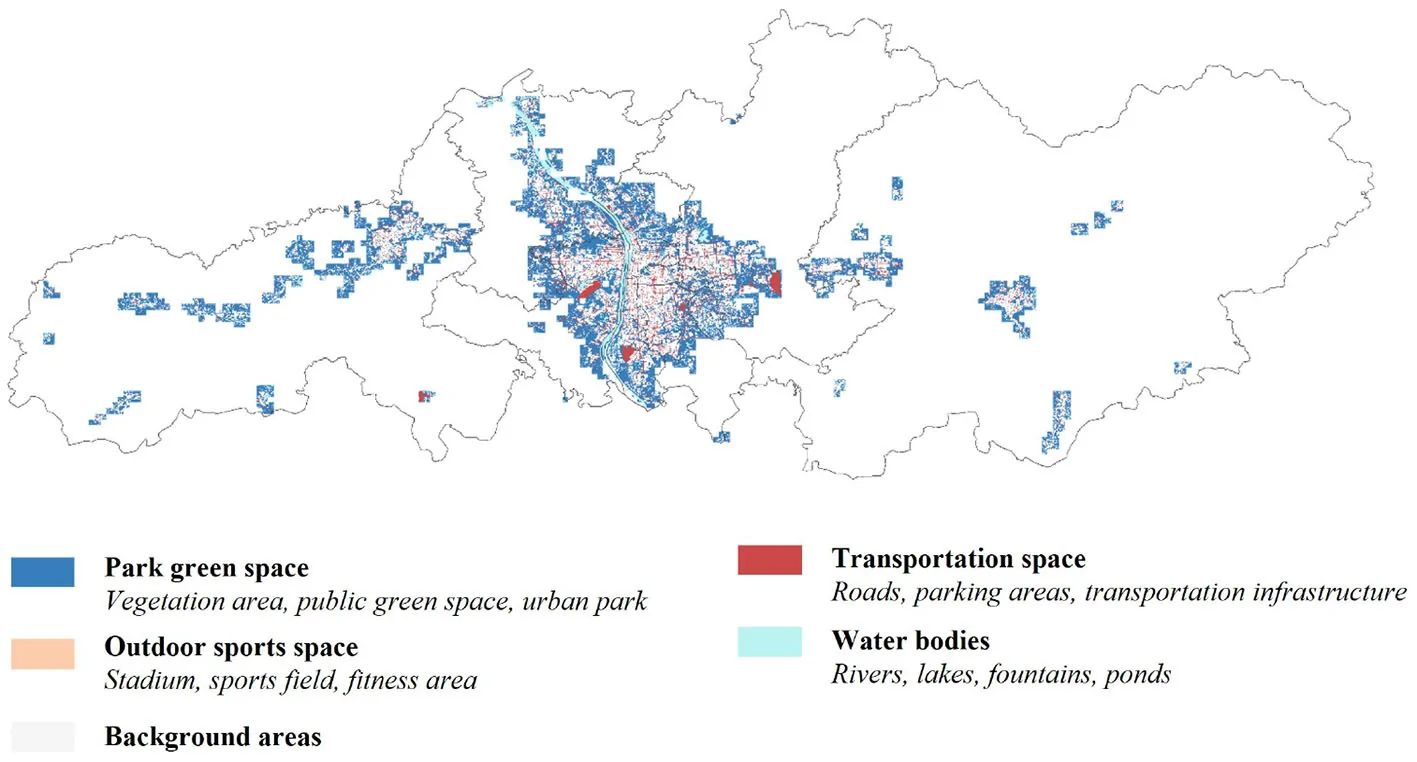
Spatial distribution and classification of public open spaces in Changsha city.

This study uses the 2021 urban open space classification dataset with a spatial resolution of 1.19 m. The dataset categorizes urban open spaces into five classes: park green space, outdoor sports space, transportation space, water bodies, and background areas (see Figure 2). As this study focuses on open and publicly accessible spaces for residents' physical activity, the “outdoor sports space” category is extracted through a reclassification process and used as the primary data source for identifying sports facilities.

The raster dataset is then vectorized in a GIS environment, converting raster cells into polygon features. Adjacent patches are merged, and topological checks are conducted to ensure data consistency. As a result, a total of 2436 sports facility patches are identified. Based on patch area, sports facilities are classified into four hierarchical levels: small facilities with an area of less than 2,000 m^2^, medium facilities with an area between 2,000 m^2^ and 10,000 m^2^, large facilities with an area between 10,000 m^2^ and 30,000 m^2^, and extra-large facilities with an area greater than 30,000 m^2^, as shown in Table 1.

**Table 1:** Classification criteria and service ranges of sports facilities.

Sports facility level	Area range (m^2^)	Service mode	Service radius
Small sports facility	< 2, 000	Walking	500 m
Medium sports facility	2, 000–10, 000	Walking	1, 000 m
Large sports facility	10, 000–30, 000	Walking/cycling	2, 000 m
Extra-large sports facility	>30, 000	Motorized transport	5 km

It should be noted that conventional fixed-buffer approaches apply the same spatial scale to all sports facilities, implicitly assuming that facilities of different levels have comparable service capacities, attraction ranges, and access modes. However, this assumption may lead to a spatial-scale mismatch and hinder a reasonable comparison of their surrounding built environments. Specifically, a relatively large fixed buffer may extend beyond the actual neighborhood-oriented service context of small facilities, whereas a relatively small fixed buffer may fail to capture the broader service context of large and extra-large facilities. Therefore, considering the differences among facility levels in terms of facility size, service capacity, attraction range, and residents' primary access modes, and with reference to previous studies and daily activity characteristics, differentiated service buffers are constructed for each facility level. Buffer distances of 500 m, 1,000 m, 2,000 m, and 5 km are applied to small, medium, large, and extra-large sports facilities, respectively. The 500 m and 1,000 m buffers represent short-distance walking catchments for community- and neighborhood-level facilities, while the 2,000 m buffer represents an extended active-travel catchment accessible by walking or cycling. The 5 km buffer captures the broader service context of extra-large facilities, which generally have stronger spatial attractiveness and greater reliance on motorized travel. Rather than treating all sports facilities as having homogeneous service ranges, this hierarchical delineation aligns the spatial scale of built environment measurement with the differentiated service functions and accessibility characteristics of each facility level, thereby providing a more reasonable basis for comparison. The delineated service areas are subsequently used as the basic spatial units for built environment measurement and spatial analysis.

## 3 Research framework and methodology

### 3.1 Data sources and quantification of the built environment

To comprehensively characterize the built environment surrounding sports facilities, this study integrates multiple spatial datasets, including road network data, POI data, remotely sensed vegetation cover data, and population distribution data. A built environment indicator system is constructed from five dimensions: accessibility, facility provision, functional structure, ecological environment, and population characteristics. Considering the heterogeneity in service capacity and travel behavior associated with different levels of sports facilities, the service coverage area of each facility is adopted as the basic analytical unit for quantifying surrounding built environment attributes.

Specifically, road density is employed to represent the connectivity and permeability of the local road network. Commercial density and sports facility density are used to capture the intensity of facility provision within the service area. The POI Mix Index is applied to measure the degree of functional and land-use diversity. The normalized difference vegetation index (NDVI) is utilized to reflect the quality of the surrounding ecological environment, while population density serves as an indicator of potential service demand. Together, these six indicators provide a comprehensive quantitative description of the built environment around sports facilities and form the basis for subsequent analyses of built environment differentiation and pattern identification across facility levels.

#### 3.1.1 Road network data

**Figure 3:**
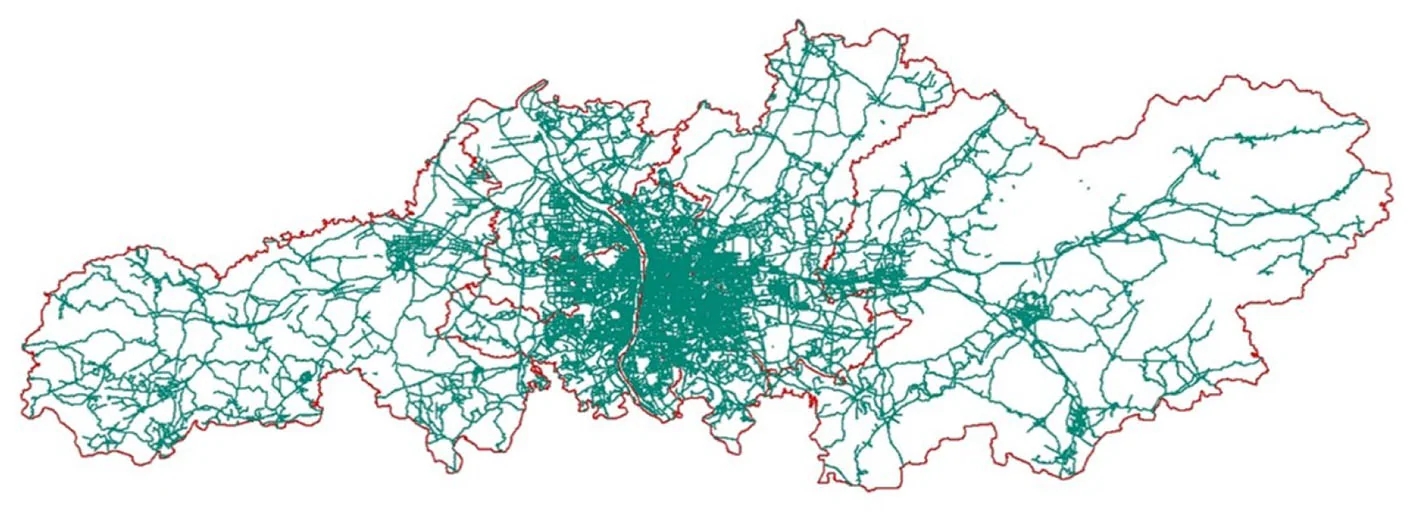
Road network distribution of Changsha city.

In this study, the road network data are obtained from OpenStreetMap (OSM), an open-source geographic data platform widely used in transportation analysis and accessibility studies (17, 18). OSM provides relatively comprehensive information on road hierarchy, network topology, and connectivity. Vector road network data covering the entire administrative area of Changsha city (see Figure 3) are acquired from the official OSM platform in April 2026.

**Figure 4:**
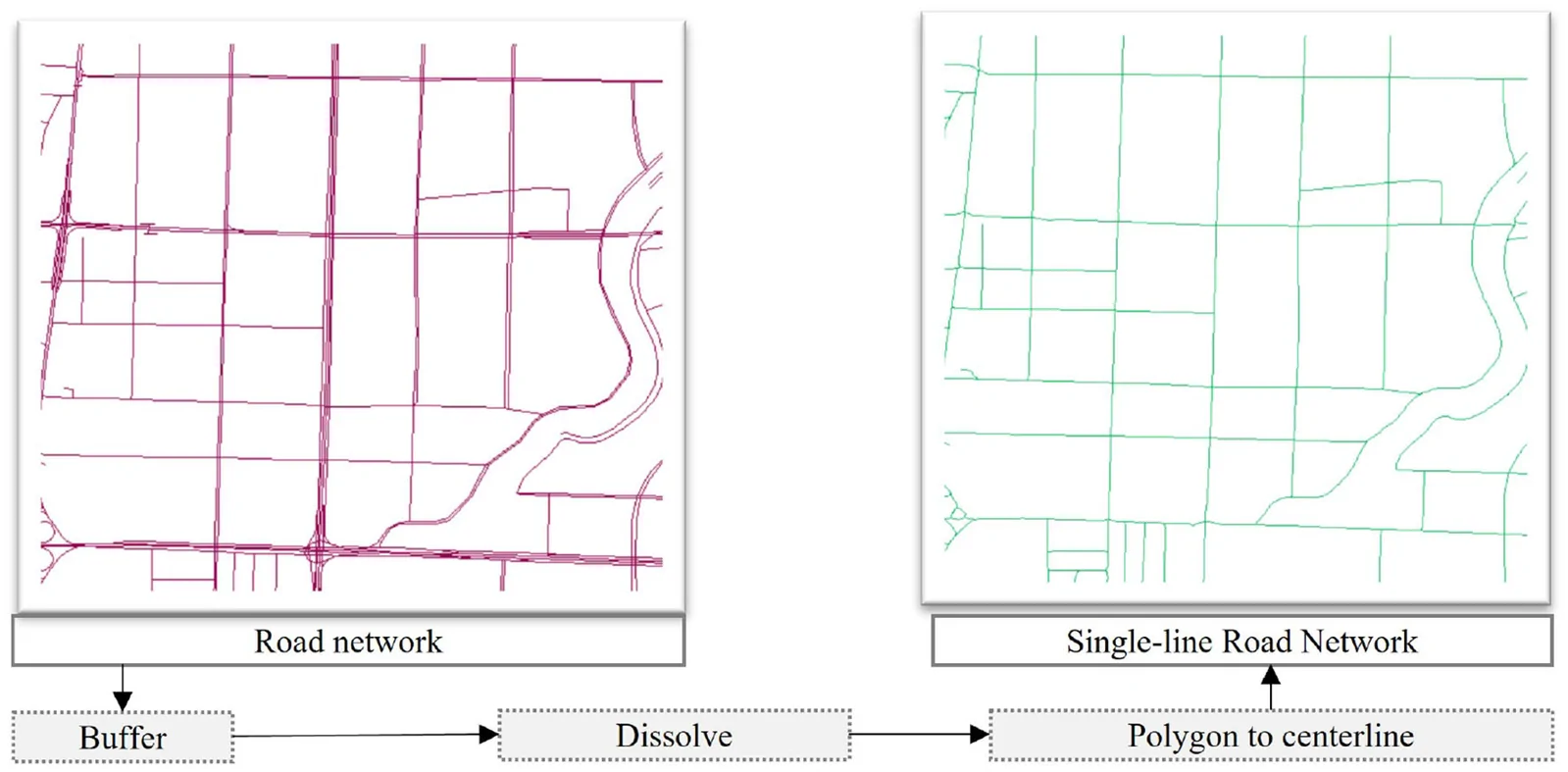
Workflow of buffer-based road density calculation.

As a volunteered geographic information product, the completeness and spatial accuracy of OSM data may vary across different areas, particularly in suburban zones and for lower-class roads (19). To improve data quality, OSM road data are cross-validated using the Amap API. Missing major roads are supplemented, while road elements irrelevant to the research objectives, such as bridle paths and roads under construction, are removed. In addition, common topological errors, including dangling nodes and duplicate line segments, are systematically corrected to ensure spatial consistency and network connectivity. The processed road network serves as the base dataset for subsequent accessibility analysis. To better capture the spatial characteristics of different travel modes, the road network is further classified into pedestrian and motorized systems and preprocessed separately. First, geometric correction and topological cleaning are applied to ensure spatial accuracy and network connectivity. Second, roads are classified according to road hierarchy. Based on the standard for classification of urban land use and planning construction land in China, original road classes are categorized into expressways, arterial roads, first-class roads, second-class roads, and third-class roads, which are further aggregated into three levels: (1) expressways and arterial roads; (2) first- and second-class roads; and (3) third-class roads. Meanwhile, buffer distances of 40 m, 20 m, and 10 m are assigned to the three road levels, respectively, to represent differences in traffic capacity and spatial extent. Buffers of different road classes are then merged to generate continuous road surface features. Subsequently, a polygon-to-centerline conversion method is applied to extract road centerlines, transforming dual-line road representations into a single centerline network. This process eliminates redundant road expressions and improves computational efficiency for network-based analysis. The whole workflow of buffer-based road density calculation is shown in Figure 4.

To quantify road network connectivity and walkability around sports facilities, road density is adopted as an accessibility indicator (20). After road network preprocessing, GIS spatial analysis is used to calculate the total length of road centerlines within the service area of each sports facility. Road density is then computed by dividing the total road length by the area of the service coverage, as expressed by the following equation:


$$\begin{array}{c} RD=\frac{L}{A} \end{array}$$ (1)

where *RD* denotes road density (km/km^2^), *L* is the total length of road centerlines within the service area (km), and *A* represents the area of the service coverage (km^2^).

#### 3.1.2 Green space data

This study employs the NDVI to quantify green space exposure within the study area. NDVI is a widely used indicator for assessing vegetation density and health conditions and is commonly applied in studies of ecological environment and environmental exposure (21, 22). The NDVI data are derived from the MODIS MOD13Q1 product provided by NASA, which offers a spatial resolution of 250 m and a temporal resolution of 16 days (23). All available images covering the growing season from April to October 2025 are collected. To represent annual vegetation conditions, a maximum value composite method is applied to generate a representative NDVI layer for the study year.

All data processing is conducted on the Google Earth Engine platform. The preprocessing steps include reprojection to the WGS84 UTM Zone 50N coordinate system, clipping to the administrative boundary of Changsha, and setting NDVI values below 0 to zero in order to exclude non-vegetated surfaces. The NDVI is calculated based on the reflectance values of the near-infrared and red spectral bands using the following formula (24):


$$\begin{array}{c} \text{NDVI}=\frac{\text{NIR}-\text{RED}}{\text{NIR}+\text{RED}} \end{array}$$ (2)

where NIR denotes the reflectance of the near-infrared band and RED denotes the reflectance of the red band. NDVI values range from −1 to 1, with higher values indicating greater vegetation coverage and higher levels of green space exposure.

#### 3.1.3 Population data

Population data are derived from the WorldPop global high-resolution population gridded dataset (25, 26) in this paper. This dataset integrates multiple sources, including remote sensing imagery, land use information, nighttime light data, and census statistics, and applies machine learning-based dasymetric modeling to spatially disaggregate population counts. It provides a reliable representation of fine-scale population distribution patterns. In this study, population data with a spatial resolution of 100 m are used to characterize the spatial distribution of residents within the study area. Since the pixel values in the WorldPop dataset represent the estimated number of people within each grid cell, the zonal statistics method is employed to calculate the total population within the service areas of different levels of sports facilities.

Considering that the service areas of sports facilities at different levels vary in spatial extent, direct comparison of total population may be biased by area effects. To control for differences in spatial scale, population density is further introduced to represent the degree of population concentration around sports facilities. It is calculated as:


$$\begin{array}{c} PD=\frac{P}{A} \end{array}$$ (3)

where *PD* denotes population density (persons/km^2^), *P* represents the total population within the service area (persons), and *A* is the area of the service area (km^2^). Population density reflects the intensity of potential service demand and the degree of population aggregation around sports facilities. A higher population density indicates a greater number of residents per unit area, suggesting stronger potential demand for sports facility services and more intensive built environment characteristics in the surrounding area (27).

#### 3.1.4 Functional data

Functional data are derived from the POI dataset obtained from the Amap (Gaode Map) open platform (28). POI data provide detailed information on the spatial distribution of urban functional facilities and serve as a key proxy for characterizing urban functional structure and land use patterns. To ensure data completeness and temporal consistency, all POI records within the study area of Changsha city are collected and subsequently cleaned, harmonized, and reclassified according to the official Amap classification system.

Given the relevance of surrounding commercial and sport-related services to the context of sports facility use, this study focuses on two major categories of POIs: commercial functions and sport-related functions. Commercial functions include five subcategories, namely catering services, retail and shopping, life services, leisure and entertainment, and accommodation services (29, 30). Sport-related functions primarily include fitness and sports facilities.

Based on the service coverage areas of sports facilities, spatial statistics are conducted for each POI category. Two indicators are calculated, namely commercial facility density and sports facility density, to represent the supply level of relevant supporting services around sports facilities. The density is calculated as:


$$\begin{array}{c} D_{i}=\frac{N_{i}}{A} \end{array}$$ (4)

where *D*_*i*_ denotes the density of facility type *i* (POIs/km^2^), *N*_*i*_ represents the number of POIs of type *i* within the service area, and *A* is the area of the service area (km^2^).

In addition to single-category density measures, this study further evaluates the diversity and mixing level of urban functions using the Shannon diversity index, which captures both the richness and evenness of different POI categories. A higher value indicates a more diverse and balanced functional environment. The POI functional mix index is calculated as:


$$\begin{array}{c} POI_{mix}=-\overset{n}{\underset{i=1}{\sum }}p_{i}\ln\left(p_{i}\right) \end{array}$$ (5)

where *POI*_*mix*_ denotes the POI functional mix index, *p*_*i*_ represents the proportion of POIs belonging to category *i*, and *n* is the total number of POI categories.

It should be noted that the POI functional mix index effectively reflects the complexity of land use and the degree of functional integration around sports facilities. Higher functional diversity generally indicates greater access to a variety of urban services within a limited spatial range and is often associated with greater neighborhood vitality and convenience of facility use (31). Therefore, commercial facility density, sports facility density, and POI functional mix index are jointly employed as key indicators to characterize the functional environment surrounding sports facilities.

### 3.2 Residents' sports facility use, environmental perception, and exercise intention survey

To descriptively characterize residents' sports facility use, physical activity participation, exercise intentions, and perceptions of sports facilities and their surrounding built environments, this study employs a structured questionnaire survey to collect behavioral and perceptual data. The questionnaire is designed to capture multiple dimensions of residents' interaction with sports facilities and their surrounding environments, including socio-demographic characteristics, facility usage behavior, perceived built environment, exercise intention, and perceived accessibility and barrier-free conditions.

Socio-demographic information is collected first, including gender, age, education level, occupation, and household income. These variables are introduced to account for individual heterogeneity and to improve the robustness of subsequent behavioral interpretation across different population groups. Focusing on actual usage behavior, the sports facility usage section records respondents' interaction patterns with sports spaces. Key variables include commonly used facility types (such as parks, community sports grounds, and fitness centers), preferred travel modes, typical travel time to facilities, and frequency of use. Together, these indicators reflect both accessibility and intensity of facility utilization in daily life. Beyond objective usage patterns, residents' subjective evaluation of their surrounding environment is captured through the perceived built environment section. Attention is given to walking convenience, richness and diversity of nearby facilities, functional complexity, environmental comfort, and overall neighborhood vitality. These perceptual dimensions help bridge the gap between measurable spatial structure and lived urban experience. From a mobility and accessibility perspective, the perceived barrier-free environment section examines walking continuity, sidewalk quality, street connectivity, traffic safety, and physical barriers. Such indicators are particularly relevant for understanding ease of access to sports facilities and for assessing inclusiveness across different user groups. Exercise intention is measured as a self-reported behavioral propensity toward physical activity participation. This construct is operationalized through multiple indicators, including exercise habits, self-reported activity level, perceived health benefits, and willingness to maintain long-term engagement. Higher scores represent stronger self-reported motivation and intention to maintain physical activity participation.

All questionnaire items are measured using a structured Likert-scale format to ensure comparability across respondents (32). Together, these items provide descriptive measures of respondents' self-reported facility use, physical activity participation, exercise intention, and environmental perceptions. These measures are used to characterize the surveyed sample rather than to estimate the causal effects of built-environment characteristics on exercise intention or physical activity.

### 3.3 Data analysis methods

To examine the differences in built environment characteristics surrounding different levels of sports facilities and their spatial distribution patterns within life circles, this study employs a combination of statistical and spatial analysis methods, including one-way analysis of variance (ANOVA) (33, 34), clustering analysis (35, 36), chi-square tests (37), and spatial analysis (38, 39).

First, one-way analysis of variance (ANOVA) is used to examine whether the mean values of the six built-environment indicators differ across the four sports facility levels. Facility level is treated as the grouping variable, while population density, commercial density, sports facility density, road density, POI functional mix index, and NDVI are examined separately as dependent variables. For indicators showing a statistically significant overall difference, Tukey's honestly significant difference post-hoc test is used to identify pairwise differences between facility levels. Statistical significance is used to determine whether the observed differences are unlikely to result from sampling variation alone, whereas the practical magnitude of the differences is interpreted by jointly considering the group means, their absolute and relative differences, and their relevance to facility planning. Therefore, a statistically significant result is not automatically interpreted as a strong practical difference.

Second, K-means clustering is applied to identify typical combinations of built-environment characteristics surrounding sports facilities. Before clustering, all six indicators are standardized using the Z-score method to remove differences in measurement units and numerical ranges. The number of clusters is determined by jointly considering the elbow method, the silhouette coefficient, and the interpretability of the resulting centroid profiles. The elbow method evaluates how the within-cluster sum of squares decreases as the number of clusters increases and identifies the point beyond which additional clusters provide only limited improvement in cluster compactness. The silhouette coefficient is used to assess within-cluster cohesion and between-cluster separation. These statistical criteria are considered together with whether the resulting clusters present distinct, non-redundant, and substantively interpretable built-environment profiles. Based on this combined assessment, four clusters are retained. The resulting categories are interpreted and labeled directly according to the dominant standardized indicator values at each cluster centroid. The category names therefore describe population density, facility and service intensity, POI functional mix index, and NDVI characteristics rather than implying actual levels of human activity, urban vitality, or recreational use.

Third, to explore the association between sports facility levels and built environment types, a contingency table is constructed, and a chi-square test (χ^2^ test) is conducted to examine whether significant relationships exist between facility levels and cluster membership distributions.

Finally, GIS-based spatial analysis is used to visualize and analyze the spatial distribution of clustering results. Spatial pattern maps are generated to illustrate the distribution of different built environment types, while standard deviational ellipse analysis is employed to examine spatial centers, dominant orientations, and dispersion ranges of each cluster. These analyses help reveal the spatial organization characteristics of built environment patterns around sports facilities.

In addition, descriptive statistical analysis is applied to questionnaire survey data (40–42). Frequency, percentage, and mean statistics are used to summarize residents' sports facility use, reported physical activity characteristics, environmental perceptions, accessibility perceptions, and exercise intentions. The questionnaire results are subsequently interpreted alongside the corresponding objective built-environment dimensions at the thematic and aggregate levels to provide complementary behavioral and perceptual context for the spatial findings. This analysis does not estimate individual-level statistical associations or causal effects between the built environment and physical activity or exercise intention.

## 4 Results and discussion

### 4.1 Scale distribution of sports facilities in Changsha city

**Figure 5:**
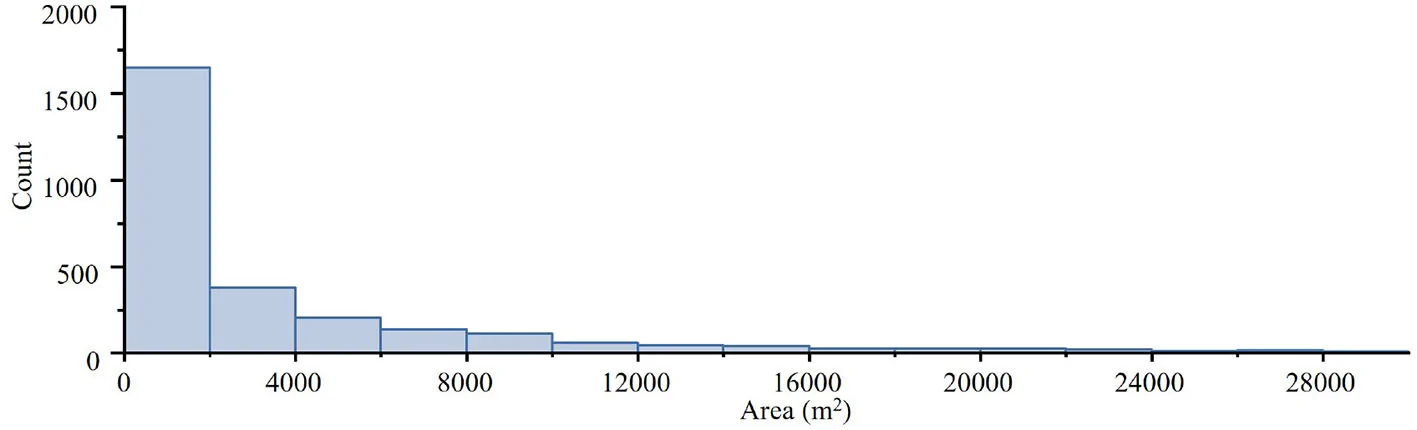
Distribution of sports facility areas in Changsha city.

A statistical analysis is conducted on the areas of 2,436 extracted sports facility patches (Figure 5), with a total area of approximately 11.33 km^2^. The results indicate that the area distribution of sports facilities exhibits a pronounced right-skewed distribution. Most facility patches are concentrated within relatively small area ranges, with small-sized facilities accounting for an overwhelming majority. As facility size increases, the number of patches decreases rapidly, and only a limited number of large and mega sports facilities are observed. The frequency distribution demonstrates a typical long-tail pattern, whereby a small number of large-scale facilities contribute disproportionately to the total area, while the majority consist of community-level, small-scale venues. This pattern reflects the spatial configuration of urban public sports facilities characterized by “a large number of small facilities coexisting with a few large comprehensive venues” and provides a critical basis for subsequent analyses of service areas and facility hierarchy.

**Figure 6:**
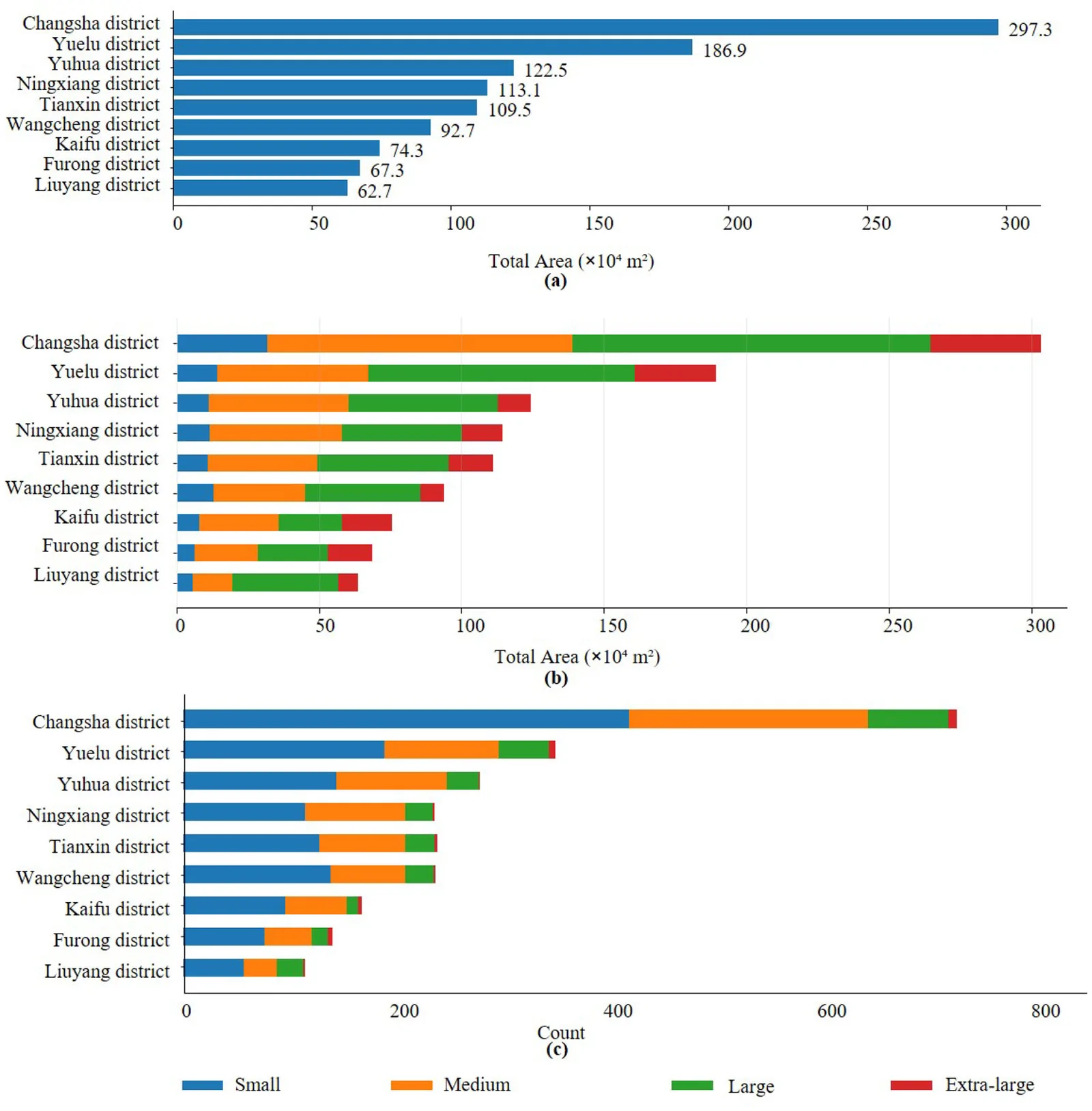
Sports facility size structure across districts of Changsha city: **(a)** total sports facility area, **(b)** facility-area composition by facility size, and **(c)** facility counts by facility size.

At the district level, sports facility resources in Changsha city display significant spatial differentiation, with substantial variations in total area, size composition, and facility counts among districts (Figure 6). In terms of total facility area, Changsha district ranks first, reaching 3.01 km^2^, accounting for approximately 26.6% of the total area in the study area. This is followed by Yuelu district (1.88 km^2^) and Yuhua district (1.23 km^2^). In contrast, Liuyang district, Furong district, and Kaifu district each have total facility areas of less than 0.80 km^2^, indicating that sports facility resources are primarily concentrated in districts with higher levels of urban development and stronger population and functional agglomeration (Figure 6a).

Regarding the facility size structure, small-sized sports facilities dominate Changsha city, totaling 1,334 facilities and accounting for 54.8% of all facilities. Medium-, large-, and extra-large facilities represent 32.6%, 11.4%, and 1.3%, respectively. Changsha district contains the largest number of facilities (711), substantially exceeding Yuelu district (342) and Yuhua district (273). Overall, all districts exhibit a consistent trend in which the number of facilities declines progressively with increasing facility size (Figure 6b).

However, the distribution of facility counts does not correspond to their area contributions. Although large-sized facilities account for only 11.4% of the total number, they occupy 4.80 km^2^, contributing 42.4% of the total facility area and constituting the primary source of spatial provision. Medium-sized facilities cover 3.88 km^2^ (34.2%), whereas the most numerous small-sized facilities contribute only 9.7% of the total area (Figure 6c). At the district scale, Changsha district records 1.25 km^2^ and 1.06 km^2^ of large- and medium-sized facility areas, respectively, while Yuelu district possesses the largest area of large-sized sports facilities citywide (0.93 km^2^). Overall, the sports facility system in Changsha city exhibits a hierarchical spatial organization, in which numerous small facilities support daily community-level activities, while a limited number of large facilities provide district- and city-level services.

This hierarchical size structure also supports the use of differentiated service areas according to facility size rather than uniform buffers or administrative units. Applying an identical analytical scale to facilities with different potential service ranges and dominant access modes could mischaracterize their surrounding built environments (43, 44). The hierarchy-sensitive delineation adopted in this study therefore provides a standardized basis for comparing the environmental contexts of different facility levels. Nevertheless, these service areas should be interpreted as proxies for potential facility catchments rather than exact representations of residents' actual travel or activity spaces.

### 4.2 Spatial patterns of built environment and sports facilities in Changsha city

**Figure 7:**
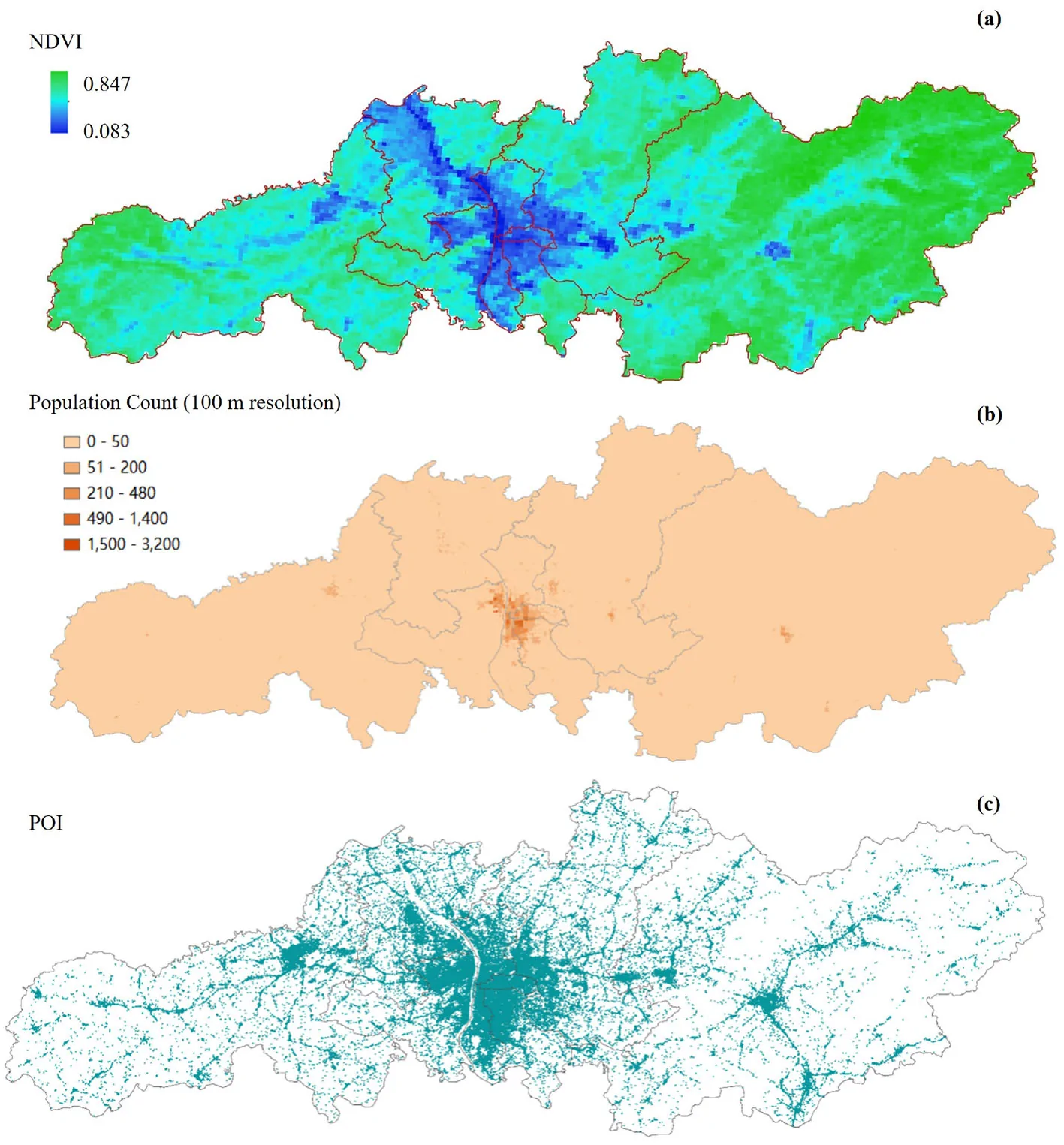
Spatial distribution of built-environment indicators in Changsha City: **(a)** NDVI, **(b)** population density (100 m resolution), and **(c)** POIs.

Figure 7 illustrates the overall spatial patterns of ecological environment, population distribution, and sports facility resources in Changsha city, revealing pronounced spatial heterogeneity and a clear center–periphery gradient across multiple spatial scales.

Regarding the ecological environment (Figure 7a), NDVI values range from (0.083) to (0.847), exhibiting a general pattern of lower values in the urban core and higher values toward peripheral areas. The central urban districts, including Yuelu district, Tianxin district, Furong district, Yuhua district, and Kaifu district, together with areas along the Xiangjiang River, show relatively low vegetation coverage, whereas Ningxiang district, Wangcheng district, Changsha district, and Liuyang district, particularly mountainous areas such as Yuelu Mountain and the Yanghu Wetland, form contiguous high-NDVI ecological zones. This pattern shows a spatial correspondence between intensive urban development and relatively low vegetation coverage in the city center.

In contrast, population distribution exhibits an opposite spatial trend (Figure 7b). High population densities are highly concentrated within the core built-up areas of Changsha city, mainly in Furong district, Yuhua district, Tianxin district, Kaifu district, and Yuelu district, where population counts in some (100, m) grids exceed (1,500) persons. Population density declines rapidly toward the periphery, particularly in Wangcheng district, Ningxiang district, Changsha district, and Liuyang district, forming a typical monocentric urban structure.

Against this background, the spatial distribution of sports facilities shows a strong population-oriented pattern (Figure 7c). Sports facilities are densely clustered within built-up areas of Changsha city and form high-density agglomerations in the urban core, while their numbers decrease markedly in peripheral districts. Compared with the NDVI pattern, the distribution of sports facilities shows a closer spatial correspondence with population distribution. Sports facilities are more concentrated in densely populated areas, although this descriptive correspondence does not establish that population concentration determines facility location. Nevertheless, in ecologically favorable peripheral districts such as Ningxiang district, Wangcheng district, and Liuyang district, sports facilities, although relatively dispersed, still form localized clusters around key township centers. Overall, the spatial distribution of sports facilities corresponds closely with population distribution, while ecological conditions show additional variation between the urban core and peripheral areas, together forming a hierarchical built-environment pattern.

**Figure 8:**
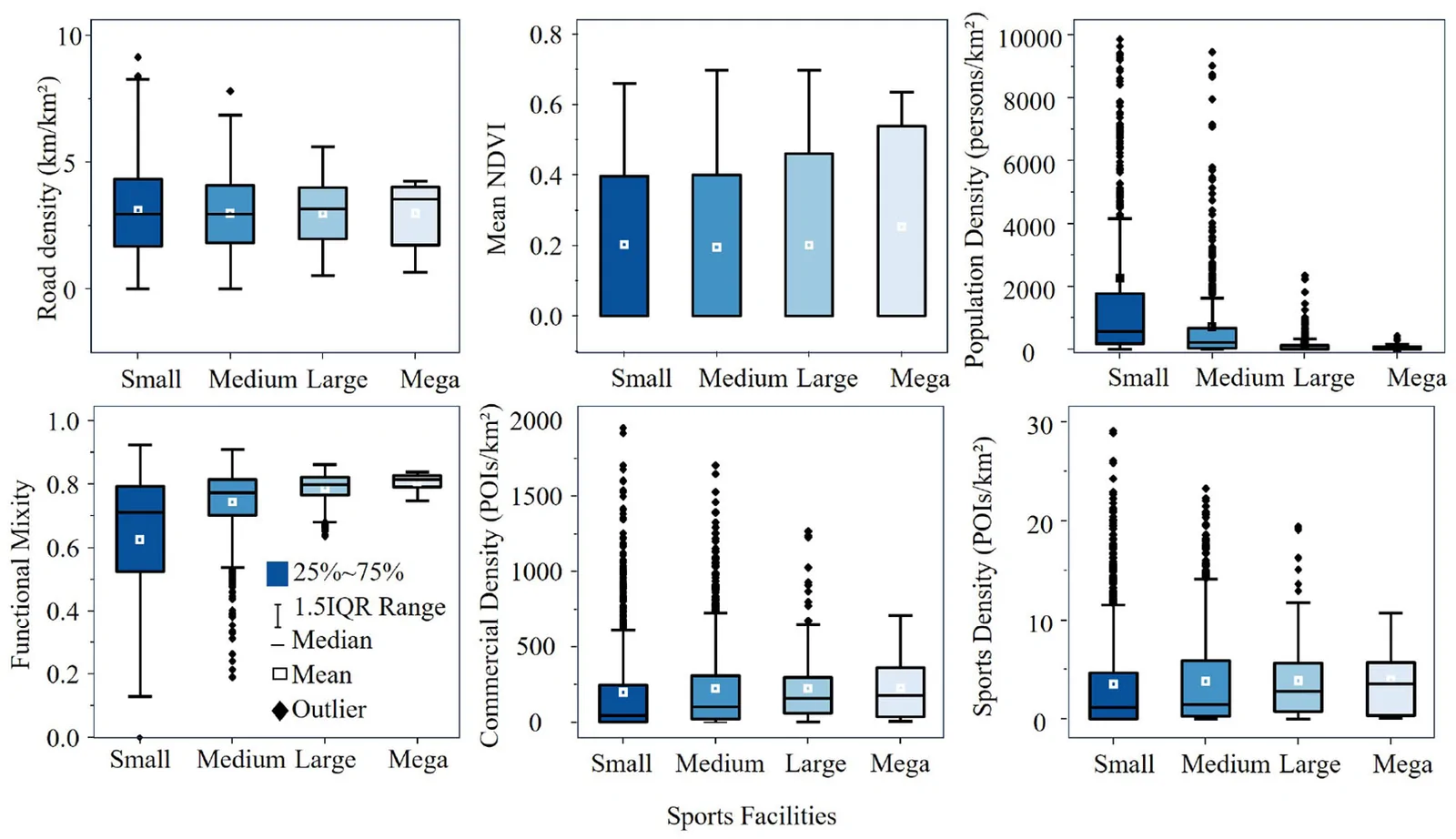
Comparison of built environment characteristics across different sports facility service areas.

Table 2 and Figure 8 further summarize the descriptive statistics of built environment characteristics across different sports facility levels. As facility level increases, surrounding built environments shift from high population density toward higher functional mix. Population density and POI functional mix index emerge as the most distinctive indicators. Small sports facilities exhibit the highest surrounding population density, with a mean of (2, 267.06, persons/km^2^), which is (3.2), (15.9), and (38.0) times that of medium, large, and extra-large facilities, respectively. The large standard deviation [(5, 382.57, persons/km^2^)] indicates that small facilities are distributed across both high-density urban neighborhoods and peripheral residential areas. In contrast, the POI functional mix index increases steadily from (0.626) to (0.807) with facility level, suggesting that large and extra-large facilities are more likely to be located in areas with diverse land-use functions and complex urban structures. Meanwhile, the standard deviation of the POI functional mix index decreases markedly from (0.228) to (0.026), indicating greater functional stability around higher-level facilities.

**Table 2:** Descriptive statistics of built environment indicators across sports facility levels.

Indicator	Small	Medium	Large	Extra-large
Population density (persons/km^2^)	2, 267.06 ± 5, 382.57	708.48 ± 1, 689.34	142.41 ± 397.26	59.65 ± 172.18
POI functional mix index (−)	0.626 ± 0.228	0.701 ± 0.114	0.763 ± 0.048	0.807 ± 0.026
Commercial density (POIs/km^2^)	228.74 ± 312.55	214.63 ± 198.42	205.17 ± 164.89	219.36 ± 142.77
Sports facility density (POIs/km^2^)	3.91 ± 5.26	3.72 ± 4.18	3.50 ± 2.96	3.63 ± 2.41
Road density (km/km^2^)	3.05 ± 0.82	3.01 ± 0.74	2.97 ± 0.69	2.99 ± 0.65
NDVI (−)	0.197 ± 0.083	0.221 ± 0.091	0.236 ± 0.088	0.254 ± 0.076

The descriptive results in Table 2 show that commercial density, sports facility density, and road density vary within relatively narrow ranges across facility levels. Commercial density ranges from approximately 205 to 229 POIs/km^2^, sports facility density ranges from 3.50 to 3.91 POIs/km^2^, and road density remains close to 3.0 km/km^2^. NDVI increases from 0.197 around small facilities to 0.254 around extra-large facilities. Although Table 3 indicates a statistically significant difference in NDVI across facility levels, the absolute difference between the group means is relatively limited compared with the pronounced gradient in population density. Therefore, the NDVI result should be interpreted as a modest cross-level difference rather than as evidence of strong ecological differentiation.

**Table 3:** One-way ANOVA results of built environment indicators across sports facility levels.

Indicator	*F*	*p*	Significance
Population density (persons/km^2^)	86.42	< 0.001	***
POI functional mix index (−)	54.17	< 0.001	***
Commercial density (POIs/km^2^)	1.38	0.247	n.s.
Sports facility density (POIs/km^2^)	0.91	0.436	n.s.
Road density (km/km^2^)	0.63	0.595	n.s.
NDVI (−)	3.94	0.008	**

The asterisks indicate the level of statistical significance.

The ANOVA results in Table 3 indicate statistically significant differences across facility levels in population density, POI functional mix index, and NDVI, whereas commercial density, sports facility density, and road density do not show statistically significant differences. However, the descriptive magnitude and planning relevance of these differences are not equivalent. Population density decreases from 2, 267.06 persons/km^2^ around small facilities to 59.65 persons/km^2^ around extra-large facilities, indicating a pronounced gradient in the population contexts surrounding different facility levels. The POI functional mix index increases from 0.626 to 0.807, showing a consistent but less pronounced gradient toward greater functional diversity around higher-level facilities. By comparison, the difference in mean NDVI is smaller, despite being statistically significant. These results suggest that population concentration and functional mix provide the clearest practical distinctions among facility levels, while the differences in the other indicators are comparatively limited.

From a planning perspective, the population-density gradient suggests that small facilities are more closely embedded in densely populated residential environments, where neighborhood-scale accessibility and everyday service demand should receive particular attention. The higher POI functional mix index around large and extra-large facilities indicates a greater need to coordinate these facilities with diverse urban functions and broader transport connections. In contrast, the relatively limited differences in commercial density, sports facility density, road density, and NDVI do not, by themselves, provide a strong basis for substantially different planning strategies across facility levels. These interpretations refer to the magnitude and spatial meaning of the observed differences and do not imply causal effects of the built environment.

The non-significant results for commercial density, sports facility density, and road density indicate only that the present analysis did not identify statistically detectable differences in their group means. They should not be interpreted as evidence of equivalent or balanced spatial provision, because such a conclusion would require additional assessments of spatial accessibility, service demand, population coverage, and distributional equity.

These findings demonstrate the value of distinguishing facility hierarchies when interpreting the built environments surrounding public services. The proposed approach may also be applicable to other public service facilities with differentiated service radii, such as parks, schools, medical facilities, and older adults care facilities (45, 46). In these applications, level-specific service areas can provide a spatial reference for 15-minute life circle evaluation, healthy city planning, public service equity assessment, and urban renewal. However, service-area thresholds should be adapted to the service capacity, dominant access mode, and local spatial context of each facility type rather than transferred mechanically.

### 4.3 Spatial differentiation of the built environment around sports facilities in Changsha city

**Figure 9:**
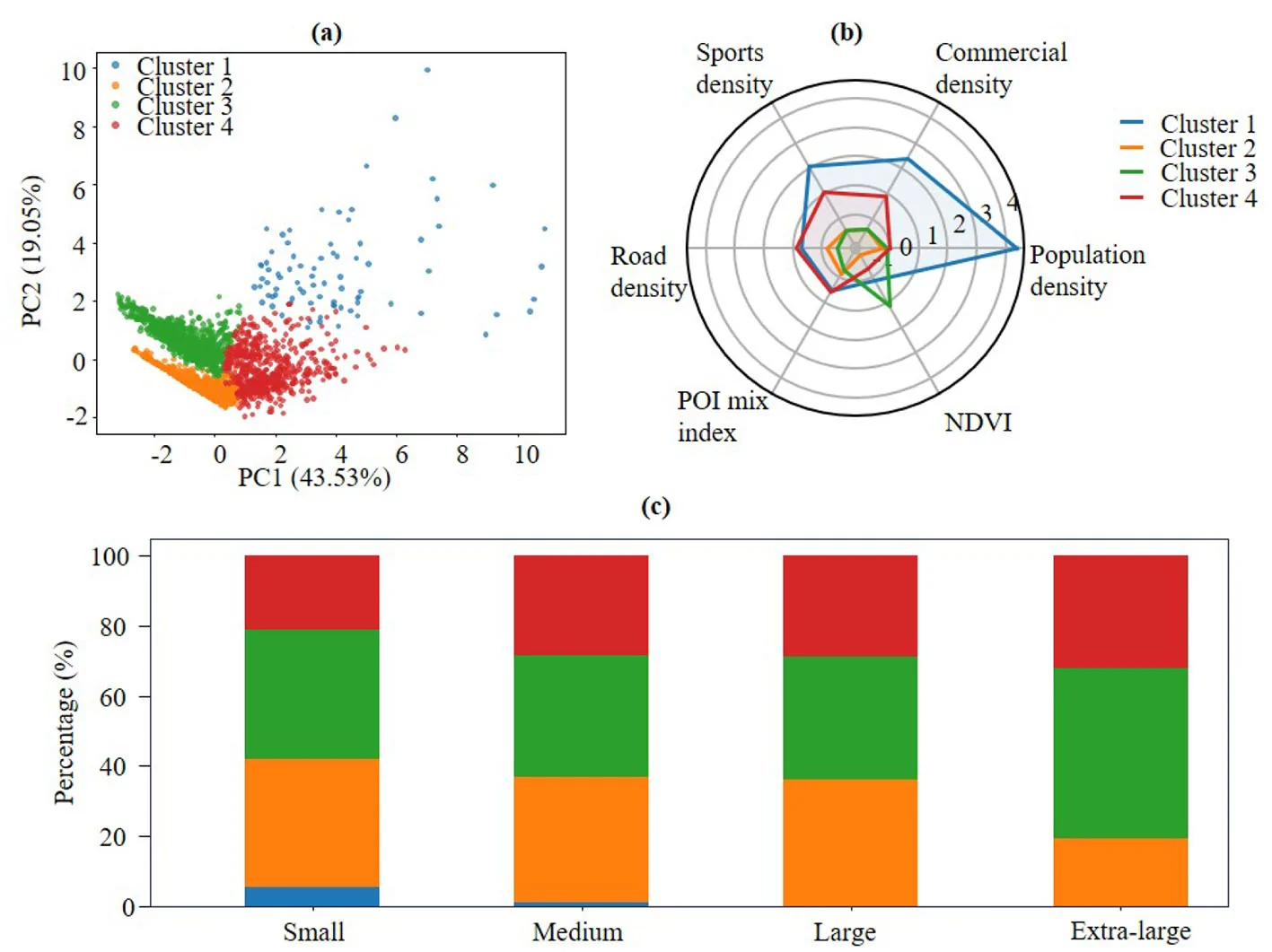
Identification of built-environment types surrounding sports facilities: **(a)** PCA visualization of K-means clustering results, **(b)** cluster centroid profiles, and **(c)** distribution of built-environment types across sports facility levels.

To identify typical built-environment configurations surrounding sports facilities, K-means clustering is applied to the six standardized built-environment indicators. The four-cluster solution is retained by jointly considering the elbow method, the silhouette coefficient, and the substantive interpretability of the resulting centroid profiles. The elbow method is used to identify the point at which increasing the number of clusters produces only limited additional reduction in within-cluster variation, while the silhouette coefficient evaluates the cohesion and separation of the candidate cluster solutions. The interpretability assessment further considers whether each cluster presents a distinct and non-redundant combination of built-environment characteristics. Based on the combined consideration of these criteria, four clusters provide a suitable balance between statistical separation and substantive interpretation. Figure 9 summarizes the identification and interpretation of the four built-environment types: Figure 9a visualizes the cluster assignments in a two-dimensional principal component space, Figure 9b presents the standardized indicator profiles of the four cluster centroids, and Figure 9c shows the proportional distribution of the four clusters across sports facility levels. Principal component analysis is used only to visualize the clustering structure rather than to determine cluster membership. In Figure 9a, each point represents the built-environment characteristics of one sports facility service area, and the colors indicate its K-means cluster assignment. The horizontal and vertical axes represent the first and second principal components derived from the six standardized built-environment indicators. The first and second principal components explain 43.53% and 19.05% of the total variance, respectively, with a cumulative explained variance of 62.58%.

The four categories are interpreted according to the relative standardized values of the six indicators represented by their cluster centroids in Figure 9b. As shown in Table 4, Cluster 1 has the highest relative levels of population density, commercial density, and sports facility density and is therefore labeled the high-density and service-intensive type. Cluster 2 has lower-than-average values across most of the measured indicators and is labeled the low-intensity built-environment type. Cluster 3 has the highest NDVI value, together with relatively low population density and POI functional mix index, and is therefore labeled the high-NDVI and low-density type. Cluster 4 has relatively high commercial density, sports facility density, and POI functional mix index and is labeled the functionally mixed and service-intensive type. These labels directly describe the dominant combinations of the measured built-environment indicators and do not imply actual levels of human activity, urban vitality, recreational use, environmental quality, or planning performance.

**Table 4:** Cluster centroids of built environment types.

Cluster	English type
C1	High-density and service-intensive type
C2	Low-intensity built-environment type
C3	High-NDVI and low-density type
C4	Functionally mixed and service-intensive type

Based on the identified built environment types, the composition of built environment patterns varies substantially across different facility hierarchies (Figure 9c). Small-scale sports facilities are mainly located within Cluster 2 and Cluster 3, accounting for (36.36%) and (37.18%), respectively, while Cluster 4 accounts for (21.06%). As facility size increases, the proportion of Cluster 4 rises steadily, indicating that higher-level sports facilities tend to concentrate in more functionally mixed urban spaces. Meanwhile, the share of Cluster 2 declines, whereas Cluster 3 maintains a relatively high proportion across all facility levels. For extra-large sports facilities, Cluster 3 becomes the dominant type (48.39%), followed by Cluster 4 (32.26%), while Cluster 2 decreases to (19.35%). In contrast, Cluster 1 appears only marginally in small and medium facilities and is nearly absent in large and extra-large facilities, suggesting that highly compact and intensely developed environments are not the primary locations for high-level sports facilities.

The chi-square test (χ^2^ = 57.71, *p* < 0.001) indicates that the distribution of built-environment types differs significantly across facility levels. However, statistical significance does not indicate that the practical differences among all facility levels are equally strong. The practical interpretation is therefore based on the observed differences in cluster proportions. In particular, the proportion of the functionally mixed and service-intensive type increases with facility level, while the low-intensity built-environment type becomes less common and the high-NDVI and low-density type accounts for a comparatively large proportion of extra-large facilities. These compositional differences indicate that higher-level facilities more frequently correspond to functionally mixed and service-intensive or high-NDVI and low-density settings, whereas small facilities occur across a wider range of built-environment contexts.

The cluster profiles provide a basis for context-sensitive planning rather than a uniform ranking of facility environments. High-density and service-intensive environments may warrant attention to pedestrian accessibility and coordination among concentrated facilities and services. Low-intensity built environments indicate relatively low values across the measured indicators, but further demand and accessibility assessments are required before these areas can be interpreted as deficient in service provision. In high-NDVI and low-density environments, planning may consider the continuity of green environments together with the accessibility of sports facilities. Functionally mixed and service-intensive environments may benefit from coordination among sports facilities, surrounding services, and multimodal transport. These implications are derived from the measured characteristics of the clusters and should be understood as planning references rather than demonstrated effects or direct evaluations of planning performance.

**Figure 10:**
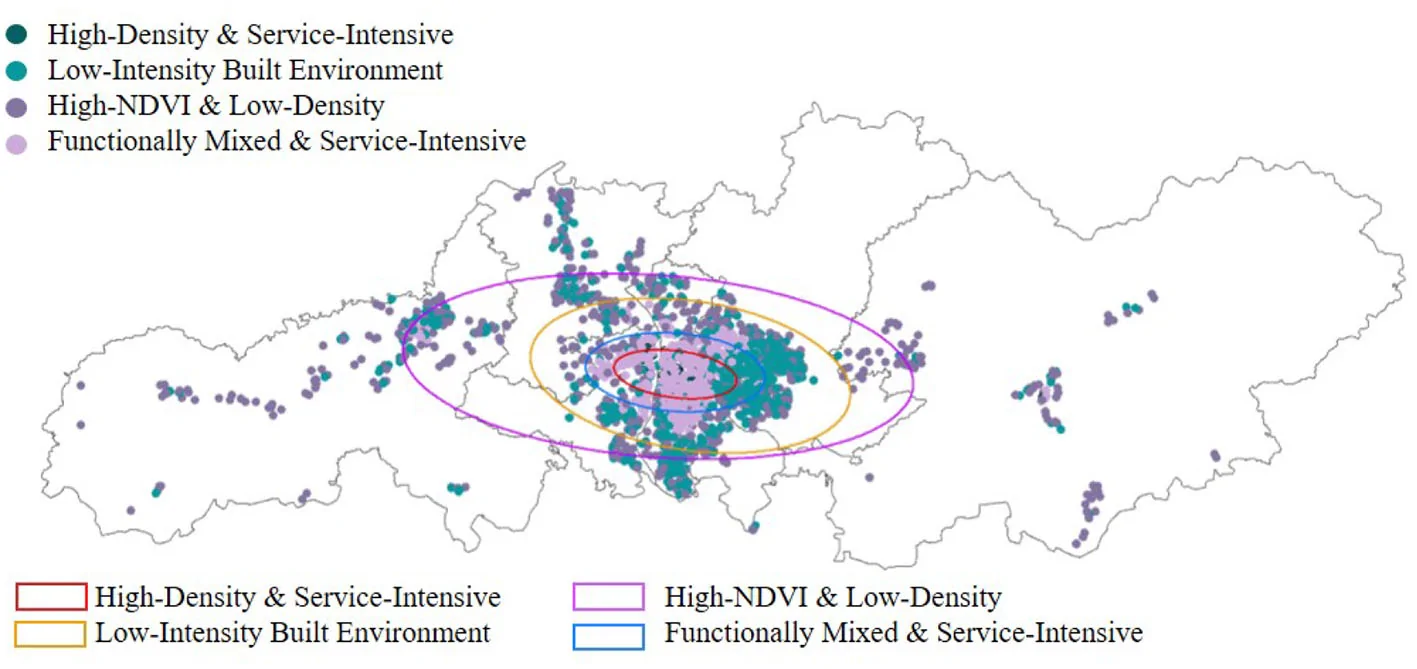
Spatial distribution and standard deviational ellipses of built-environment types surrounding sports facilities.

Figure 10 illustrates the spatial distribution of the four built environment types across Changsha city and their corresponding standard deviation ellipses. All types exhibit a clustered distribution centered on the urban core, while displaying substantial differences in spatial extent and organizational structure. The high-density and service-intensive type is concentrated in the central urban area and has the smallest ellipse area of 14.93 km^2^. The low-intensity built-environment type has a considerably larger spatial extent, with an ellipse area of 124.18 km^2^, and gradually extends toward peripheral urban areas. The high-NDVI and low-density type has the largest spatial dispersion, with an ellipse area of 240.38 km^2^, and is widely distributed across Ningxiang district, Wangcheng district, and Liuyang district, often forming belt-shaped patterns along ecological corridors. The functionally mixed and service-intensive type has a moderate spatial extent of 36.27 km^2^ and is mainly concentrated in Yuelu district, Tianxin district, Furong district, Yuhua district, and Kaifu district, extending toward newly developed urban areas.

Further analysis indicates that the major axes of the standard deviation ellipses for all four types are oriented approximately east-west, with rotation angles ranging from (94°) to (99°), closely aligned with the Xiangjiang River development axis and the overall spatial expansion direction of Changsha city. This alignment shows a close spatial correspondence between the built-environment patterns surrounding sports facilities and the city's macro-spatial structure.

### 4.4 Characteristics of sports facility use and physical activity based on questionnaire survey

To strengthen the integration of the spatial and questionnaire analyses, the questionnaire results are interpreted according to the same built-environment dimensions used in the objective assessment. Reported travel modes, travel times, walking convenience, and route continuity are considered in relation to the accessibility dimension; perceptions of facility functions and surrounding services are considered alongside facility density and POI functional mix index; and the use and perception of natural activity spaces are discussed in relation to NDVI and the high-NDVI and low-density type identified through clustering. Residents' facility-use frequency and activity-space choices provide complementary behavioral context for these dimensions. However, because the questionnaire responses are not spatially linked to specific facilities or individual-level objective indicators, this integration represents an aggregate-level comparison rather than a statistical test of association or causality.

**Figure 11:**
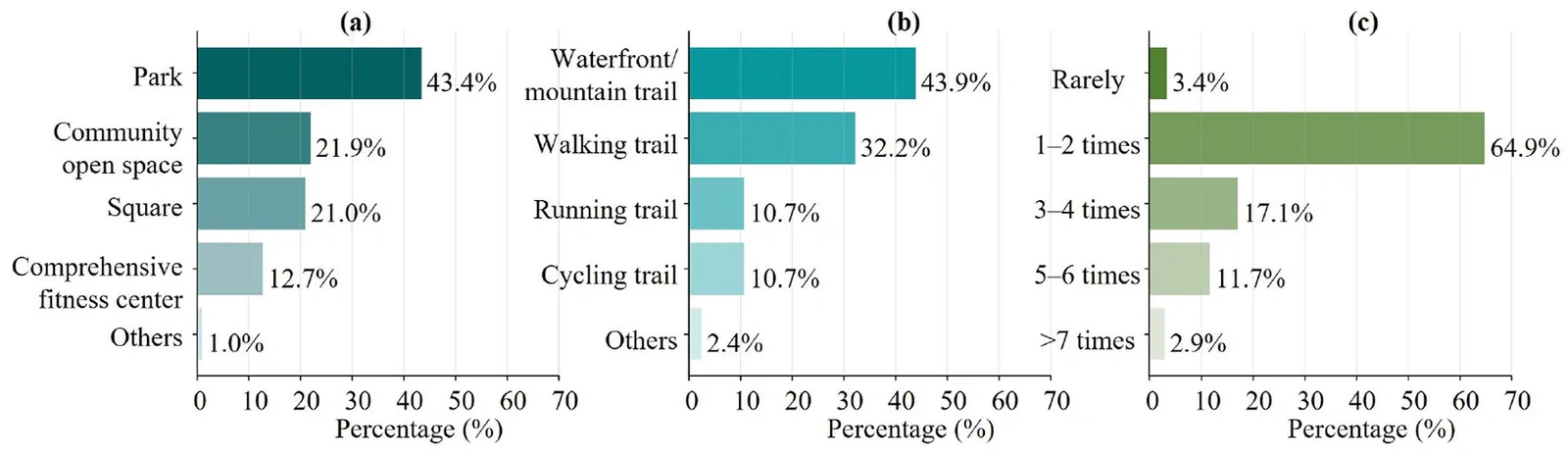
Characteristics of sports facility use: **(a)** types of frequently used activity spaces, **(b)** types of frequently used trails, and **(c)** frequency of sports facility use.

Figure 11 illustrates the overall characteristics of sports facility use among residents in Changsha city, highlighting clear patterns in spatial preference, accessibility, and participation frequency. Overall, residents' physical activity behavior demonstrates a pronounced preference for open public spaces and a strong community-oriented pattern. Regarding the types of activity spaces, parks are the most frequently used venues, accounting for 43.41%, followed by community open spaces (21.95%) and public squares (20.98%). In contrast, comprehensive fitness centers and other facility types exhibit substantially lower usage rates. These results show that respondents most frequently used open and relatively low-threshold public spaces for daily physical activity. This behavioral pattern provides complementary context for the objective finding that numerous small facilities are embedded within community environments. In terms of specific activity settings, waterfront and mountain trails show the highest utilization rate (43.90%), followed by walking trails (32.20%), both of which have higher reported usage rates than running and cycling trails (each 10.73%). The reported preference for waterfront, mountain, and walking trails provides complementary behavioral context for the high-NDVI and low-density type identified through spatial clustering and for the relatively high NDVI values surrounding some higher-level facilities. Nevertheless, this aggregate-level correspondence does not demonstrate that NDVI or other ecological conditions determined respondents' activity-space choices. With respect to usage frequency, sports facility participation is dominated by moderate-frequency participation. The proportion of residents using facilities 1–2 times per week reaches 64.88%, whereas those exceeding 5 times per week account for less than 15%. Thus, weekly participation was commonly reported, whereas high-frequency facility use was less common. Further descriptive analysis of travel mode and accessibility reveals that walking and running constitute the primary means of access (60.00%), followed by cycling (29.76%), while public transport and private cars play a minor role. Moreover, more than 85% of respondents can reach their commonly used sports facilities within 15min. The predominance of active travel and short reported travel times provides behavioral context for the walking- and cycling-oriented service ranges adopted in the hierarchical spatial framework. However, these results describe the experiences of the surveyed residents and do not independently verify the service radius of each facility level.

The predominance of walking and cycling and the generally short reported travel times are broadly consistent with previous studies emphasizing the importance of proximity and active-travel accessibility in the use of neighborhood physical-activity facilities (47, 48). In the present study, these behavioral findings complement the objective accessibility dimension and support the practical relevance of differentiating service areas by facility hierarchy. Nevertheless, because the questionnaire responses are not linked to particular facility levels, this correspondence should be interpreted as contextual evidence rather than as direct validation of the adopted buffer distances.

**Figure 12:**
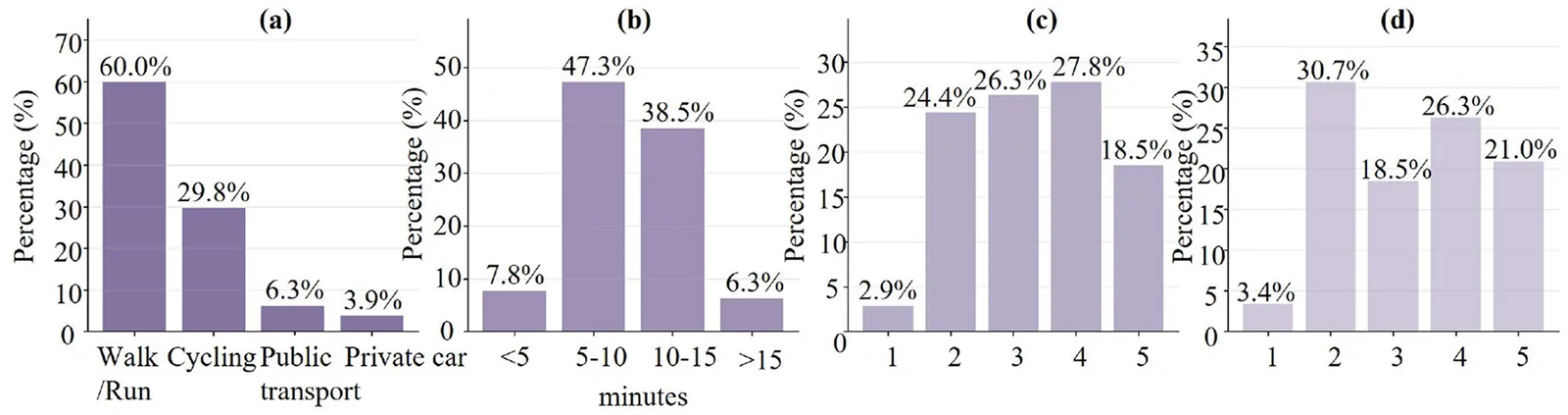
Travel characteristics and perceived walking accessibility related to sports facility use: **(a)** travel mode, **(b)** travel time, **(c)** walking convenience, and **(d)** route continuity and barrier-free accessibility.

Figure 12 illustrates respondents' travel characteristics to sports facilities and their subjective evaluations of the walking environment. A five-point Likert scale (1–5) is adopted in the questionnaire, where 1 indicates “very dissatisfied” and 5 indicates “very satisfied.” Overall, respondents reported relatively short travel times and generally moderate-to-positive perceptions of the walking environment around the sports facilities they used. In terms of travel mode choice, residents primarily rely on active modes to access sports facilities. Walking or running accounts for 60.00%, while cycling represents 29.76%, together comprising nearly 90% of all trips. This distribution is broadly consistent with the role of walking and cycling in the differentiated service-area framework. Correspondingly, travel time to sports facilities is generally short, with 5–10min and 10–15min accounting for 47.32% and 38.54%, respectively, totaling more than 85%. This indicates that most respondents reported reaching their commonly used facilities within 15min, but it does not establish that all facilities have equivalent accessibility. Ratings of walking convenience are mainly concentrated between scores of 3 and 5, with 4 and 5 together accounting for 46.34%. Similarly, evaluations of route continuity and barrier-free accessibility are dominated by medium-to-high scores, with 4 and 5 comprising 47.32%, clearly exceeding the proportion of low ratings. These subjective assessments complement the objective finding that road density varies relatively little across facility levels, suggesting that a broadly similar level of basic road-network provision coexists with generally moderate-to-positive perceptions of walking accessibility. Because road density and questionnaire responses are not matched at the respondent level, this comparison should be interpreted as contextual consistency rather than evidence that road density affects travel time, walking convenience, or physical activity frequency.

**Figure 13:**
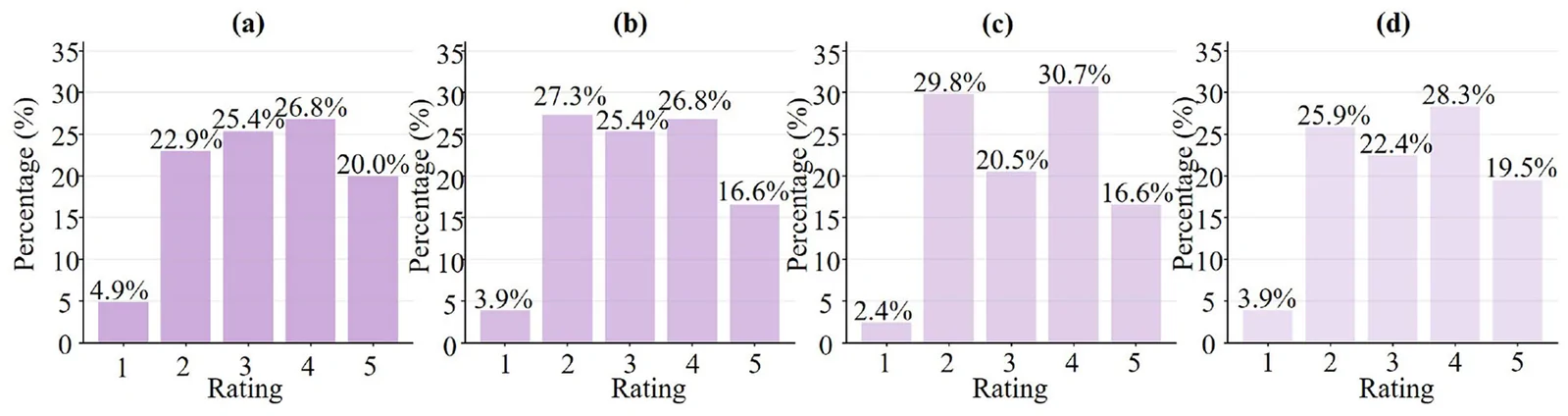
Respondents' evaluations of sports facilities: **(a)** functional richness, **(b)** ramp accessibility, **(c)** facility occupation during use, and **(d)** facility pricing.

Figure 13 presents residents' subjective evaluations of sports facilities in terms of functional configuration, barrier-free environment, usage conditions, and pricing. A five-point Likert scale is employed in the questionnaire, where 1 indicates “very dissatisfied” and 5 indicates “very satisfied,” with a maximum score of 5. In terms of functional provision, ratings are mainly concentrated at scores of 3 and 4, with 4 accounting for the largest share (26.83%), indicating generally moderate-to-positive perceptions of facility functions. Regarding accessibility and barrier-free design, evaluations of ramp design and circulation convenience are dominated by medium-to-high scores, with 4 and 5 together accounting for 43.41%, reflecting generally moderate-to-positive perceptions of barrier-free conditions. As evaluations extend to usage conditions, perceptions of facility occupation show greater variability. Scores of 2 and 4 account for 29.76% and 30.73%, respectively, indicating heterogeneous perceptions of facility occupation and congestion. Finally, residents express relatively high satisfaction with pricing, as scores of 4 and 5 together reach 47.80%, showing that nearly half of the respondents provided positive evaluations of facility pricing. The generally moderate evaluations of facility functions provide a subjective counterpart to the objective results for sports facility density, while the variability in facility occupation indicates that the amount of spatial provision alone may not fully describe residents' experiences of facility availability. However, no direct statistical relationship between objective facility density and these subjective evaluations is tested in the present study.

**Figure 14:**
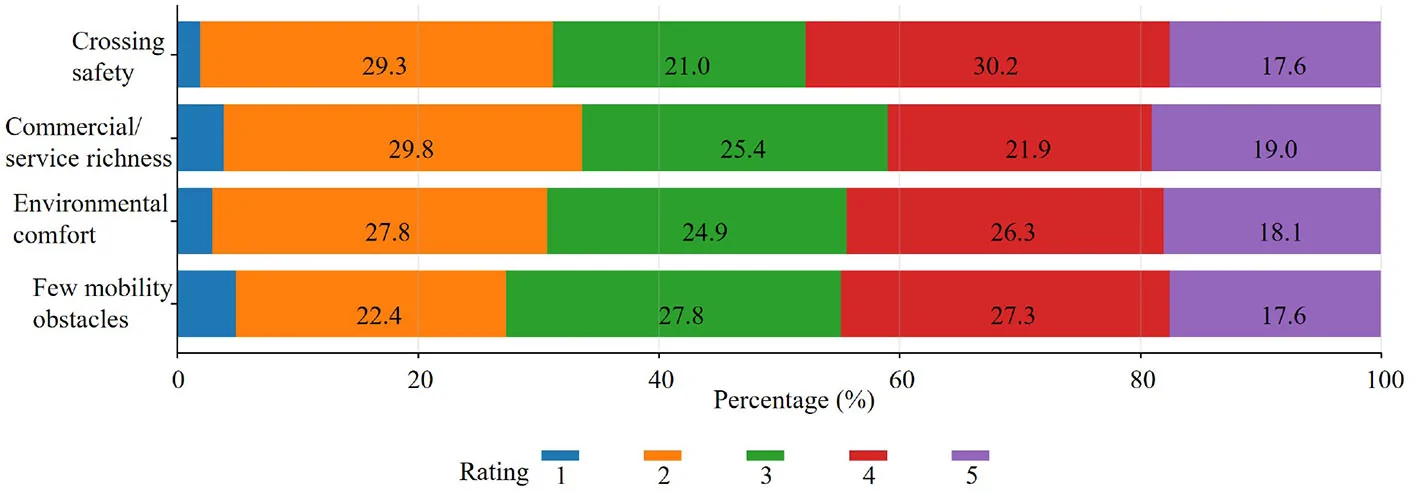
Distribution of respondents' ratings of built-environment and pedestrian conditions surrounding sports facilities.

Figure 14 illustrates residents' perceived evaluation of the built environment and pedestrian accessibility surrounding sports facilities. A five-point Likert scale (1–5) is adopted in the questionnaire, where 1 indicates “very dissatisfied” and 5 indicates “very satisfied.” Overall, respondents expressed a generally positive perception of the surrounding environment, with most ratings concentrated at scores of 3 and 4, indicating generally moderate-to-positive subjective evaluations of the environments surrounding the facilities they used. Among the evaluated dimensions, crossing safety received the highest level of approval, with ratings of 4 and 5 accounting for 47.8% of responses. This shows that respondents generally perceived the traffic organization and pedestrian crossing conditions around the facilities they used as relatively safe. In addition, perceptions of commercial and service richness as well as environmental comfort are also dominated by medium-to-high ratings. These two perceptual dimensions correspond conceptually to the objective POI functional mix index, commercial density, and NDVI indicators. The generally favorable perception of surrounding services provides subjective context for the functional characteristics identified by the spatial analysis, while perceived environmental comfort complements the ecological dimension represented by NDVI. These correspondences are interpretive rather than statistical because the subjective ratings are not linked to objective indicator values for the facilities used by individual respondents. As the evaluation further extended to pedestrian environment quality, perceptions of few mobility obstacles showed a more differentiated distribution. Although ratings of 3 and above still accounted for the majority, a relatively higher proportion of 2-point ratings indicates that some respondents perceived obstacles in the surrounding pedestrian environment. Such perceptions may relate to conditions such as illegal parking, sidewalk encroachment, or suboptimal spatial arrangements, but their specific causes are not independently verified by the present survey.

Overall, the integrated interpretation shows that the objective and subjective results describe complementary aspects of the environment–activity context. The spatial analysis identifies population density and POI functional mix index as the principal indicators differentiating facility levels and distinguishes high-density and service-intensive, low-intensity built-environment, high-NDVI and low-density, and functionally mixed and service-intensive environments. The questionnaire results add information on how respondents used and perceived sports facilities, including their preferences for open and natural activity spaces, reliance on walking and cycling, generally short travel times, and evaluations of accessibility, functional provision, and environmental comfort. The reported use of active travel is contextually consistent with the walking- and cycling-oriented service framework; the use of waterfront and mountain trails provides complementary behavioral context for the high-NDVI and low-density pattern, and perceptions of surrounding service richness provide subjective context for the observed POI functional characteristics. Nevertheless, because the two datasets are integrated at the conceptual and aggregate levels rather than through respondent-level spatial matching, these findings do not demonstrate that the objective built-environment indicators are statistically associated with or causally influence physical activity. Future research should link geocoded questionnaire responses and specific facility-use records with objective built-environment indicators to test these relationships directly.

## 5 Conclusions

This study develops a built-environment analysis framework based on sports facility hierarchy by integrating remote sensing imagery, road networks, POI data, population data, and questionnaire survey data. By defining differentiated service areas according to facility size, the framework provides a hierarchy-sensitive basis for characterizing the built environments surrounding different facility levels. The results show that population density and POI functional mix index provide the clearest practical differentiation among facility levels. Small sports facilities are primarily embedded in high-density residential communities, whereas large and extra-large facilities are more frequently located in areas with higher functional mix and slightly higher mean NDVI. Commercial density, sports facility density, and road density exhibit comparatively limited cross-level variation, but their non-significant differences do not indicate equivalent or balanced spatial provision. Cluster analysis identifies four descriptive built-environment patterns: high-density and service-intensive, low-intensity built-environment, high-NDVI and low-density, and functionally mixed and service-intensive types. The questionnaire results further show that respondents predominantly use open and natural activity spaces, rely mainly on walking and cycling, generally report short travel times, and provide moderate-to-positive evaluations of accessibility, facility functions, surrounding services, and environmental comfort. These behavioral and perceptual findings complement the objective spatial results at the thematic and aggregate levels.

Meanwhile, several limitations should be acknowledged. First, the sports facility data are derived mainly from urban open-space datasets and focus on outdoor activity spaces; therefore, indoor venues and some commercial sports facilities are not fully represented. Second, the classification thresholds and level-specific service radii constitute operational spatial-scale choices. Although they provide a standardized basis for comparing facilities with different potential service ranges, alternative thresholds or buffer distances may produce different aggregated indicator values, cross-level differences, and cluster assignments. The present study does not systematically test the sensitivity of the results to alternative spatial scales. Accordingly, the differentiated service areas should be interpreted as standardized proxies for potential facility catchments rather than exact representations of residents' actual travel or activity spaces. Third, the cross-sectional design provides a snapshot of sports facilities, surrounding built environments, and residents' reported behaviors and perceptions. It cannot identify temporal changes, determine the direction of the observed relationships, or establish causal effects of the built environment on physical activity. Fourth, the objective environmental indicators characterize aggregate spatial conditions but cannot fully explain individual physical activity behavior, which may also be associated with personal preferences, socioeconomic characteristics, perceived safety, facility quality and management, actual travel routes, and other unmeasured factors. Although the spatial and questionnaire findings are jointly interpreted through corresponding accessibility, functional, and ecological dimensions, the questionnaire responses are not spatially matched to specific facilities or individual-level objective built-environment indicators. Their integration is therefore conducted at the thematic and aggregate levels and does not establish individual-level statistical associations or causal relationships. Future research could conduct multi-scale sensitivity analyses, use longitudinal or panel data, and integrate geocoded questionnaire responses, specific facility-use records, mobile trajectory data, and network-based accessibility measures. Appropriate multilevel and inferential models could then be applied to examine these relationships more directly.

Overall, covering Ningxiang district, Wangcheng district, Yuelu district, Tianxin district, Furong district, Yuhua district, Kaifu district, Changsha district, and Liuyang district, this study characterizes the spatial differences and typological patterns of the built environments surrounding different levels of sports facilities and jointly interprets them with residents' reported facility-use behaviors and perceptions. The findings demonstrate the analytical utility of a differentiated, facility-level-based service-area framework and provide an integrated spatial, behavioral, and perceptual reference for sports facility layout optimization, 15-minute life circle construction, healthy city initiatives, and context-sensitive public service planning.

## Data Availability

The raw data supporting the conclusions of this article will be made available by the authors, without undue reservation.
